# Distribution of alien animal species richness in the Czech Republic

**DOI:** 10.1002/ece3.4008

**Published:** 2018-04-02

**Authors:** Radek Gebauer, Jan Divíšek, Miloš Buřič, Martin Večeřa, Antonín Kouba, Bořek Drozd

**Affiliations:** ^1^ Faculty of Fisheries and Protection of Waters South Bohemian Research Center of Aquaculture and Biodiversity of Hydrocenoses University of South Bohemia in České Budějovice Vodňany Czech Republic; ^2^ Department of Geography Faculty of Science Masaryk University Brno Czech Republic; ^3^ Department of Botany and Zoology Faculty of Science Masaryk University Brno Czech Republic; ^4^ Department of Environmental Geography Institute of Geonics The Czech Academy of Sciences Brno Czech Republic

**Keywords:** alien species, animal, distribution pattern, Europe, habitat invasibility, invasion level

## Abstract

Biogeographical barriers formed by natural forces over billions of years have been substantially disrupted by human activity, particularly in recent centuries. In response to these anthropogenic changes, global homogenization of biota is observed at an ever‐increasing rate, causing environmental and economic losses as well as emerging health risks. Identifying factors underlying alien species richness is essential for prevention of future introductions and subsequent spread. In this study, we examined the effects of environmental and human‐related factors on distribution of alien animal species richness in the Czech Republic (Central Europe). We compiled a set of maps showing the level of invasion of six categories of alien animal species in each of 628 grid cells (*ca*. 12.0 × 11.1 km) covering the Czech Republic. Relationships between alien species richness and 12 variables characterizing climatic conditions, topography, land cover, and human population size were calculated using the generalized least squares method. Species richness of all alien species, of invertebrates, and of terrestrial species showed the strongest positive relationship with mean annual temperature, while the number of black and grey (proposed prominent invaders) and aquatic species was most closely related to the presence of large rivers. Alien vertebrates showed a strong negative relationship with annual precipitation. The highest alien animal species richness was found in and near large population centers and in agricultural landscapes in warm and dry lowlands. The gateways for alien aquatic species are rather large rivers over sport fishing and aquaculture import. Compiled maps create a powerful visual communication tool, useful in development of programs to prevent future introductions.

## 1 INTRODUCTION

Biological invasions, consequential biodiversity loss, and ecosystem function alterations are major components of human‐induced global change (Vitousek, D'Antonio, Loope, Rejmánek, & Westbrooks, 1997) and generate economic costs associated with commodity and service losses as well as with eradication and mitigation of alien species (Pimentel, 2011; Vilà et al., 2010). Serious human health risks have emerged in tandem with new introductions (Mazza, Tricarico, Genovesi, & Gherardi, 2014). Although Europe is considered to be a more frequent donor than recipient of alien species (Lambdon et al., 2008), several evidences proved that Central Europe possesses crucial historical, biogeographical, and anthropogenic predispositions for successful biological invasions (Hulme, 2007; Pyšek, Sádlo, & Mandák, 2002). Moreover, Genovesi, Carnevali, and Scalera (2015) reported that one of five threatened species in Europe is directly affected by alien species. Additionally, the accelerating growth of the global economy and international trade goes hand in hand with increase in invasion rate (Hulme, 2009; Meyerson & Mooney, 2007) with no sign of saturation (Seebens et al., 2017). Understanding the drivers and prediction of invasion processes, including determining high‐risk areas, is therefore of critical importance for designing appropriate management interventions.

The level of invasion varies along environmental gradients and among habitats (Chytrý et al., 2008; Richardson & Pyšek, 2006; Vicente, Alves, Randin, Guisan, & Honrado, 2010). Human‐made and/or disturbed habitats are generally considered more susceptible to colonization and spread of alien species and also play a key role as corridors for new invasions (Pyšek, Chytrý, Pergl, Sádlo, & Wild, 2012). Economic and demographic variables reflecting the intensity of human impact on species and habitats include propagule pressure, pathways of introduction, eutrophication, and intensity of anthropogenic disturbance and may directly influence the outcome of invasions (Patoka et al., 2016; Perdikaris, Kozák, Kouba, Konstantinidis, & Paschos, 2012). Alien species richness therefore often positively correlates with human density and activity (McKinney, 2001; Stohlgren et al., 2006), and these variables have been suggested to be more important than environmental conditions, climate, or native species richness (Pyšek, Jarošík, et al., 2010; Spear, Foxcroft, Bezuidenhout, & McGeoch, 2013). However, even regions of low human impact are not resistant to invasion (Deutschewitz, Lausch, Kühn, & Klotz, 2003; Pyšek, Genovesi, Pergl, Monaco, & Wild, 2013; Wu et al., 2010). Aforementioned variables are important components of predictions of future invasions and alien species distribution modeling because the establishment of an alien is driven by climate and land use match (Ficetola, Thuiller, & Miaud, 2007; Thuiller et al., 2005).

In this study, we aimed to investigate which factors are important in explaining alien species richness in the Czech Republic. We considered six categories of alien species. Specific objectives of this study were to provide country‐wide maps of alien animal species richness, exploring whether the above‐mentioned groups exhibit different spatial patterns, and to assess which factors best correlate with their spatial distribution.

## 2 METHODS

### 2.1 Species distribution data

Spatial distribution (presence/absence records) of alien animal species in the Czech Republic was derived from maps published by Mlíkovský and Stýblo (2006). As primary sources, they used published records in peer‐reviewed as well as in nonpeer‐reviewed journals, abstract books, books, dissertations, grey literature, web databases, and personal and correspondence communications. We extracted all alien animals with compiled map, whereas only documented recent occurrences were considered. Uncertain occurrences and those presumably vanished were excluded.

**Figure 1:**
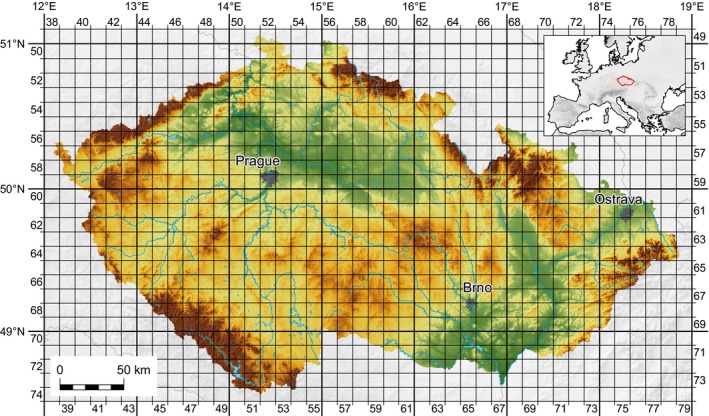
KFME grid used for mapping of the alien animal species in the Czech Republic. Three largest cities of the country are also shown

This dataset utilizes the Kartierung der Flora Mitteleuropas (KFME) mapping grid, a commonly applied mapping system in Central Europe (Buchar, 1982). The KFME is based on rectangular spatial units (grid cells). Each grid cell spans 10′ of longitude and 6′ of latitude, which represents an area of *ca*. 12.0 × 11.1 km (133.2 km^2^) on the 50th parallel (Figure 1). Although the area of the Czech Republic (78,866 km^2^) is covered by 678 grid cells in total, we excluded marginal ones and considered only 628 cells according to Šťastný, Bejček, and Hudec (2006).

For analyses of alien animal species richness, six alien animal groups were defined based on the mentioned dataset:

1. all alien animal species (207 species);
2. black and grey species, that is, prominent invasive alien species of high concern that are the subject of priority monitoring and management (Pergl et al., 2016) for the country (36 species);
3. vertebrates (58 species);
4. invertebrates (149 species);
5. terrestrial species (either vertebrate or invertebrate, 158 species);
6. aquatic species (either vertebrates or invertebrates, dependent on the aquatic environment at least for one phase of life history, 49 species).

For a list of species in each category, see Supplementary Information.

### 2.2 Explanatory variables

We calculated 12 variables describing environmental conditions and human population in each grid cell (Table 1) using ArcGIS (v. 10.2.2, ESRI, Redlands, CA, USA). Mean altitude was based on the digital elevation model of the Czech Republic with a resolution of 50 × 50 m. Based on this model, we also estimated terrain heterogeneity using the Vector Ruggedness Measure (VRM; Sappington, Longshore, & Thompson, 2007). The VRM combines variation in slope and aspect into a single measure and provides more complete information about terrain heterogeneity than do indices based on slope or altitude only. Higher VRM value indicates the higher terrain heterogeneity.

**Table 1:** Environmental variables (values per KFME mapping grid cell) used

Variable	Minimum	Mean	Maximum	*SD*
Altitude (m asl)	154	452	1,156	172
Terrain heterogeneity (VRM × 1,000)	0.0	5.3	34.3	5.4
Mean annual temperature (°C)	3.0	7.4	9.5	1.1
Annual precipitation (mm/year)	432	678	1,387	157
Proportion of forested landscape (%)	0.0	33.2	98.0	18.1
Proportion of open and mosaic landscape (%)	0.4	17.1	55.6	11.1
Proportion of arable land (%)	0.0	40.3	90.8	23.7
Number of land cover types	3	10.6	19	2.5
Proportion of (semi‐)natural habitats (%)	0.3	7.0	64.2	8.0
Proportion of water bodies (%)	0.0	1.0	17.1	1.8
Size of rivers (index)	2	4.7	8	1.3
Human population (Number of inhabitants)	1	16,534	604,751	33,944

Mean annual temperature and precipitation were extracted from maps published by Tolasz et al. (2007).

Relative area of three broad land cover types within each grid cell was calculated using CORINE 2000 Land Cover data (Bossard, Feranec, & Otahel, 2000). To obtain relative area of forests, we amalgamated the CORINE‐type *broad‐leaved forests* (3.1.1.), *coniferous forests* (3.1.2), and *mixed forests* (3.1.3). Relative area of open and mosaic landscape was calculated based on the following categories: *vineyards* (2.2.1), *fruit tree and berry plantations* (2.2.2), *pastures* (2.3.1), *complex cultivation patterns* (2.4.2), *land principally occupied by agriculture, with significant areas of natural vegetation* (2.4.3), *natural grasslands* (3.2.1), and *moors and heathland* (3.2.2). Relative area of arable land was expressed as *nonirrigated arable land* (2.1.1). In addition to the relative area of the land cover types, we calculated the number of CORINE types per grid cell in order to quantify landscape heterogeneity. Proportion of (semi‐)natural habitats was obtained from the database of natural and semi‐natural habitat types of the Czech Republic, established during the extensive habitat mapping project coordinated by the Nature Conservation Agency of the Czech Republic 2001–2004 (Härtel, Lončáková, & Hošek, 2009). The database contains spatial information on 127 (semi‐)natural habitat types defined by Chytrý, Kučera, Kočí, Grulich, and Lustyk (2010) and mapped at a scale of 1:10,000.

Each grid cell was also characterized by the relative proportion of water bodies (lakes, reservoirs, ponds, rivers) obtained from the digital base of water management (DIBAVOD) database (http://www.dibavod.cz/). In order to quantify the size of rivers in each grid cell, we assigned a Strahler's stream order value to each segment in a river network (Strahler, 1952). Each grid cell was then characterized by the maximum Strahler's stream order value in the grid cell.

Human population size in each grid cell was calculated based on the GEOSTAT 2011 population‐grid dataset (http://ec.europa.eu/eurostat/web/gisco/geodata/reference-data/population-distribution-demography), which contains the number of inhabitants per km^2^ for the entire European Union in 2011.

### 2.3 Statistical analyses

Species richness of each considered alien group was normalized using a logarithmic transformation. Where necessary, we also log‐transformed explanatory variables [terrain heterogeneity, proportion of (semi‐)natural habitats, proportion of water bodies, and human population]. To explore trends in alien species richness, we first plotted the number of species in each grid cell against each explanatory variable. The shape of the relationship was estimated using a locally weighted polynomial regression with a smoothing span α = 0.75 (Cleveland, Grosse, & Shyu, 1991). Observed trends suggested that a linear model is an acceptable approximation of the relationships.

To test the effect of each variable on alien species richness, we used the normal error generalized least squares (GLS) method, appropriate in situations in which observations of the response variable are not independent, that is, significantly spatially correlated (spatial autocorrelation was tested using Moran's *I* statistics). The GLS fits spatial covariance among observations (*i.e.,* grid cells) to take spatial autocorrelation into account. Longitudinal and latitudinal cell centroid values were used as variables to describe spatial correlation structure within the dataset. Different models of spatial structure (spherical, Gaussian, linear and exponential; see Legendre & Legendre, 2012) were tested, and the best fitting model (exponential in all cases) was selected using the Akaike information criterion. The significance of the effect of each explanatory variable was tested using the Wald test with a critical significance level of 0.05. We use the term “effect” to indicate a statistical relationship, not proven mechanistic causation (Hawkins, 2012). All models were implemented in nlme package (Pinheiro, Bates, DebRoy, & Sarkar, 2013) in R software (R Core Team, 2016).

## 3 RESULTS

Thirty‐four alien species were present in more than 100 of the 628 analyzed cells. Of these, 21 species were accidentally introduced, 11 intentionally introduced, and two by spontaneous spread. Eleven species were distributed throughout the country.

**Figure 2:**
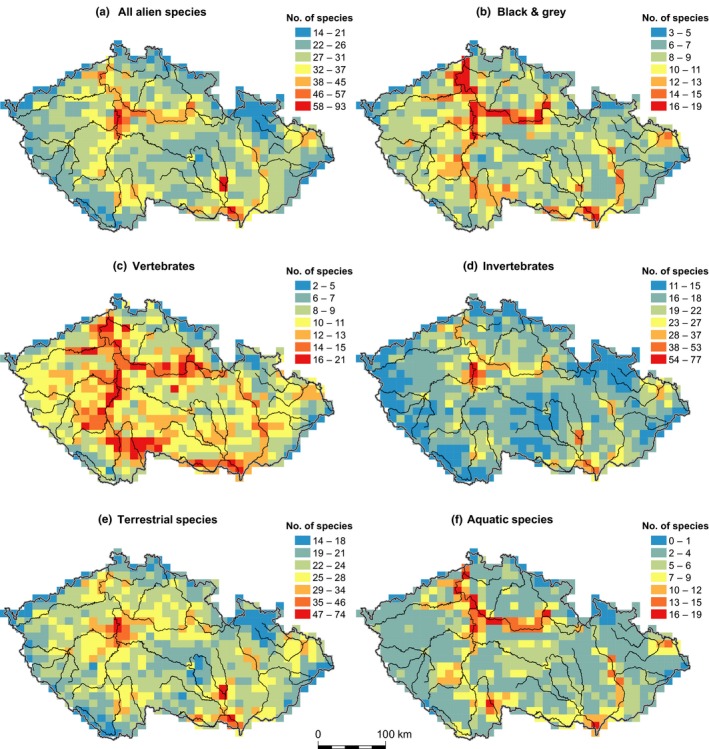
Spatial distribution of alien animal species richness in the Czech Republic. Color scale in each map was established using the Natural Breaks (Jenks) method. Thin black lines represent main river courses

**Figure 3:**
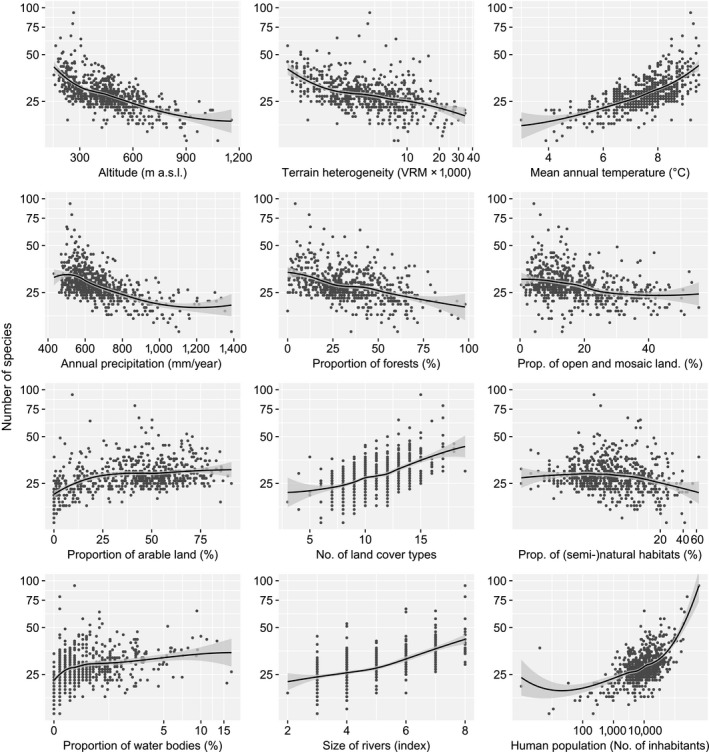
Relationships between the number of all alien animal species occurring in each grid cell (log scale) and the explanatory variables. Terrain heterogeneity, proportion of (semi‐)natural habitats, proportion of water bodies, and human population were logarithmically transformed. Regression curves were fitted using locally weighted polynomial regressions. Dark grey zone either side of the regression line denotes 95% confidence interval

In general, the highest alien animal species richness in the country was found in cities and their surroundings, floodplains of large rivers, and agricultural landscapes in warm and dry lowlands (Figure 2). Relationships among the number of all alien animal species and explanatory variables are shown in Figure 3. Using GLS, we found a significant relationship (*p *<* *.05) between species richness of each alien group and most explanatory variables (Table 2). The proportion of (semi‐)natural habitat types in grid cells was significantly negatively related to the number of alien vertebrates and showed no relationship with other groups. All alien animal species, invertebrates, and terrestrial species showed the strongest positive relationships with mean annual temperature, while the numbers of black and grey and aquatic species were best explained by river size. For vertebrates, the strongest relationship was with decreasing annual precipitation.

**Table 2:** Results of GLS models with exponential spatial structures for each considered alien animal group

	All alien species		Black & grey		Vertebrates		Invertebrates		Terrestrial species		Aquatic species	
Altitude	235.9	−−−	96.9	−−−	144.6	−−−	190.1	−−−	163.0	−−−	153.0	−−−
Terrain heterogeneity	37.9	−−−	24.5	−−−	38.5	−−−	21.3	−−−	17.8	−−−	42.0	−−−
Mean annual temperature	**278.6**	**+++**	112.5	+++	161.9	+++	**205.2**	**+++**	**188.1**	**+++**	171.7	+++
Annual precipitation	132.6	−−−	75.7	−−−	**192.1**	−−−	57.1	−−−	92.5	−−−	118.3	−−−
Proportion of forests	44.5	−−−	16.4	−−−	26.8	−−−	35.1	−−−	36.4	−−−	28.7	−−−
Proportion of open and mosaic landscape	17.9	−−−	11.1	−−−	5.5	−−	19.9	−−−	19.9	−−−	3.7	n.s.
Proportion of arable land	12.5	+++	9.6	++	25.1	+++	4.9	++	11.0	+++	9.7	++
Number of land cover types	199.5	+++	90.3	+++	66.5	+++	195.6	+++	137.3	+++	121.4	+++
Proportion of (semi‐)natural habitats	0.4	n.s.	0.0	n.s.	5.3	−	0.1	n.s.	0.0	n.s.	3.3	n.s.
Proportion of water bodies	48.7	+++	39.1	+++	52.4	+++	23.4	+++	7.9	++	107.4	+++
Size of rivers	217.4	+++	**186.4**	**+++**	119.4	+++	155.2	+++	110.1	+++	**173.4**	**+++**
Human population size	233.4	+++	88.7	+++	112.0	+++	192.8	+++	181.9	+++	142.2	+++

*F*‐values and associated significance resulting from Wald tests are shown. The highest *F*‐values are in bold. Mathematical signs + and − indicate either significant positive or negative relationships, n.s. indicates nonsignificant result. The number of symbols indicates the level of significance; +++/−−− = *p* < 0.001, ++/−− = 0.001 ≥ *p* < 0.01, and +/− = 0.01 ≥ *p* < 0.05.

## 4 DISCUSSION

Rather than attempting to disentangle pure and shared effects of environmental variables on species richness patterns, we assessed the magnitude of the relationship between alien species richness and individual factors (Table 1), producing results easily accessible to policy makers and stakeholders, as well as the public, and thus more applicable in invasion ecology management. The statistical analysis was supported by the maps of alien species richness, a powerful tool for visual communication among concerned parties, as well as for identifying invasion hotspots, crucial for prevention of future introductions, which is more effective than mitigation or eradication (Figure 2). Although newly introduced species are not included in our study, as the dataset of Mlíkovský and Stýblo (2006) has not been updated, and the distribution range of aliens, particularly those highly invasive, can expand rapidly over a short time period, it is likely that our results are currently valid, reflecting successful introductions in the past decades.

### 4.1 Topographical factors

Areas at high altitude and spatially heterogeneous terrain are generally considered to be more resistant to invasion, due to lower human population density and trade and more severe climate (Kumar, Stohlgren, & Chong, 2006; Zefferman et al., 2015). On the other hand, invasion by particular taxonomic groups, especially mammals, is largely shaped by intentional introductions and releases (Genovesi, Bacher, Kobelt, Pascal, & Scalera, 2009), which may counteract the altitudinal pattern.

We found both altitude and terrain heterogeneity to be negatively correlated with alien animal species richness. Altitude can be considered a primary determinant of climatic harshness, propagule pressure, and invasion history, factors reported to directly affect alien species richness along the altitude gradient (e.g., Konvicka, Maradova, Benes, Fric, & Kepka, 2003; Pyšek et al., 2012). This was reflected in our results showing the effect of altitude to be similar to that of mean annual temperature, precipitation, and human impact (population, land cover types), for the categories all alien species, terrestrial species, and invertebrates (Table 2). The effect of terrain heterogeneity on alien species richness was relatively weak compared to other variables and was consistently negative, suggesting that a rugged landscape with many topographical barriers may be more resistant to invasion (Richardson & Pyšek, 2006).

### 4.2 Climatic factors

Lambdon et al. (2008) identified climate as a primary driver of invasion success of alien plants. In other taxonomic groups, the effect of climate on aliens was often outweighed by demographic and economic variables such as human population density and affluence (Pyšek, Jarošík, et al., 2010). However, several studies have shown that alien mammals (Winter et al., 2010) and birds (Dyer et al., 2017) are also limited by climatic conditions. In the Czech Republic, alien animal richness was strongly positively related to the mean annual temperature and negatively related to annual precipitation. This relationship was apparent in all tested animal groups (Table 2). In invertebrates, terrestrial, and all alien species, mean annual temperature showed the strongest effect among the explanatory variables. The source of this similarity among groups is probably the high proportion of invertebrate taxa within the investigated dataset, representing 72% and 83% of all species and terrestrial species, respectively. Alien insects and invertebrates tend to occur in intensively disturbed or human‐made habitats and settlements (Pyšek, Bacher, et al., 2010). Such habitats also represent important corridors, as dispersal capability of invertebrates is often limited.

Annual precipitation and mean annual temperature were found to have the strongest effect on alien vertebrate species richness (Table 2). This result is most likely driven by correlations of annual precipitation with other variables. In the Czech Republic, precipitation is generally lower in lowlands, which are in turn warmer. The richness pattern of black and grey species was affected by climate to a lesser extent than other groups, as indicated by considerably lower *F*‐values of both climatic variables, although still significant. This underlines the adaptability and overall hardiness of prominent invaders included in the black and grey species list (Pergl et al., 2016).

### 4.3 Landscape and habitat factors

Habitat and its heterogeneity highly influence species coexistence (Chesson, 2000) and, consequently, biological invasions (Richardson & Pyšek, 2006). A heterogeneous environment provides closely linked diverse habitats suitable for both native and alien species, and therefore, they are considered more vulnerable to invasion compared to homogeneous habitats (Davies et al., 2005). This phenomenon is also supported by a positive relationship between native and alien species richness, which often occurs in large sampling areas with high habitat variability (Melbourne et al., 2007), such as the grid cells used in our study. In this study, the number of land cover types positively correlated with the species richness of all alien groups (Table 2). Considerably lower, although significant, effects were found for both vertebrates and black and grey species, probably resulting from the high taxon diversity within these groups. Moreover, vertebrates are more evenly distributed among habitats compared to other alien taxonomic groups (Pyšek, Bacher, et al., 2010), which, in our study, was reflected by their lower correlation with both proportion of forestation and proportion of open and mosaic landscape compared to that found for invertebrates.

Habitats differ in alien species richness, what is particular in alien plants (Chytrý, Jarošík, et al., 2008). Although land cover types analyzed in our study were broad heterogeneous landscape categories, they represented a range of disturbance and habitat function. The proportion of arable land reflects the man‐made cultivated habitats that are among the most invaded by plants (Chytrý, Maskell, et al., 2008). Fluctuations in available resources and severe habitat disturbance create open ecological niches suitable for aliens (Marvier, Kareiva, & Neubert, 2004). Similar trends are apparent in alien invertebrates (Pyšek, Bacher, et al., 2010; Roques et al., 2009), supported by our finding of a positive relationship of alien invertebrate richness to the proportion of arable land, although the correlation was weaker than observed for other alien species groups. The proportion of open and mosaic landscape reflected man‐made as well as natural habitats with limited available resources. Their vulnerability to invasion depends primarily upon the propagule pressure (Chytrý, Jarošík, et al., 2008), and the number of species significantly decreased with the increasing proportion of open and mosaic landscape for all alien groups except aquatic species.

The proportion of forests did not accurately reflect the human disturbance pattern across the landscape, as both semi‐natural forests and commercial plantations were included. However, most forests presumably comprise less disturbed habitats, with a low level of accessible resources compared to non‐forest habitats. This was reflected in the significant negative correlation of proportion of forests with the number of alien animals for all groups. Considering low disturbance of (semi‐)natural habitats, less invasion of these habitats might be expected. This hypothesis was confirmed only for vertebrates, other alien categories did not show significant results (Table 2). Chytrý et al. (2009) demonstrated that semi‐natural habitats in lowlands, especially those located in floodplains of large rivers, are frequent recipients of alien biota.

River size showed a significant positive relationship with all alien animal groups (Table 2). Its effect was the strongest not only on aquatic species but also on black and grey species. In the latter group, river size considerably outweighed other variables, including proportion of water bodies, due to large proportion of aquatic animals, including *Mustela vison*,* Myocastor coypus*, and *Ondatra zibethicus*, which are frequently found at river banks. Alien aquatic species in the Czech Republic show clear trends in distribution toward navigable rivers influenced by ballast water exchange, such as the Elbe River, which was recently confirmed as the site of several introduction events (Buřič, Bláha, Kouba, & Drozd, 2015). Other vectors of aquatic invasions are spontaneous migration through the Danube River system, intentional and unintentional introductions into aquaculture areas, and accidental escapes from pet owners and/or vendors (Musil, Jurajda, Adámek, Horký, & Slavík, 2010; Patoka et al., 2016).

### 4.4 Demographic factors

Propagule pressure substantially influences the level of biological invasion (Lockwood, Cassey, & Blackburn, 2005). Remote areas with little human intervention receive fewer alien species than densely populated trade routes or areas of intense human activity (Drake et al., 1989). Propagule pressure can be quantified using human‐related data as surrogate. We used human population size as an in direct indicator of human activity related to invasions. Despite limitations, this simplification is reasonable, as human population density appears to be a reliable indicator of propagule pressure in Europe (Copp, Vilizzi, & Gozlan, 2010).

When analyzed together, economic and demographic indicators have been shown to considerably outperform climatic, geographic, and land cover factors in richness of several taxonomic groups (Dyer et al., 2017; Pyšek, Jarošík, et al., 2010). In our study, human population density showed a strong positive relationship to alien animal species richness in all assessed categories (Table 2). Its considerably higher effect on invertebrates, and consequently terrestrial and all alien animals, likely reflects a high number of alien invertebrates introduced as pests into commodity storehouses. Deliberate stocking of animals within the aquatic, black and grey, and vertebrate species categories (García‐Berthou et al., 2005) into remote areas is another important factor reducing the correlation with human population density.

## 5 CONCLUSIONS

Our results together with created maps contribute to better understanding and prediction of introductions of alien animals. This information is of critical importance for managers and policy makers so they can concentrate their efforts on high‐risk areas. Moreover, they should also focus on raising the public awareness about alien species. Compiled maps represent a valuable communication tool for this purpose.

The distribution of introduced aliens, as well as establishment of new ones, also calls for further monitoring, as distribution ranges can change substantially due to the time‐lag between introduction and spread (Roques et al., 2009) as well as changes of climate (Dukes & Mooney, 1999) and land use (Sala et al., 2000). In addition, increasing temperatures can promote the invasion and reproduction success of alien species that may subsequently become established or naturalized (Walther et al., 2009).

## CONFLICT OF INTEREST

None declared.

## AUTHOR CONTRIBUTION

A.K. and M.B. conceived the original idea and developed the theory. R.G. and B.D. carried out the data acquisition. J.D. and M.V. performed the computations, analyses, and interpretation of the data. R.G. wrote the manuscript with support of all authors. All authors discussed the results and contributed to the final manuscript, revising it critically for important intellectual content.

## DATA ACCESSIBILITY

Data with numbers of alien animal species in grid cells are openly available in Dryad.
