# Supplementary material for: Distribution of alien animal species richness in the Czech Republic
Source: Ecol Evol. 2018 Apr 2;8(9):4455–64. doi: 10.1002/ece3.4008 (PMC5938441; doi:10.1002/ece3.4008)
Supplement: Supplementary file 1 [file ECE3-8-4455-s001.docx]

**Distribution of alien animal species richness in the Czech Republic**

Radek Gebauer^1^, Jan Divíšek^2,3,4^, Miloš Buřič^1^, Martin Večeřa^3^, Antonín Kouba^1^, Bořek Drozd^1^

^1^ University of South Bohemia in České Budějovice, Faculty of Fisheries and Protection of Waters, South Bohemian Research Center of Aquaculture and Biodiversity of Hydrocenoses, Zátiší 728/II, 389 25 Vodňany, Czech Republic

^2^ Department of Geography, Faculty of Science, Masaryk University, Kotlářská 2, 611 37 Brno, Czech Republic

^3^ Department of Botany and Zoology, Faculty of Science, Masaryk University, Kotlářská 2, 611 37 Brno, Czech Republic

^4^ Department of Environmental Geography, Institute of Geonics, The Czech Academy of Sciences, Drobného 28, 602 00 Brno, Czech Republic

Running title: Species richness of alien animals

Correspondence

Radek Gebauer University of South Bohemia in České Budějovice, Faculty of Fisheries and Protection of Waters, South Bohemian Research Center of Aquaculture and Biodiversity of Hydrocenoses, Zátiší 728/II, 389 25 Vodňany, Czech Republic

E-mail: [rgebauer@frov.jcu.cz](mailto:rgebauer@frov.jcu.cz)

Tel.: +420 728 586 119

**SUPPLEMENTARY INFORMATION**

List of alien animal species in particular groups (in Latin, alphabetically ordered). Letters after the Latin name denote prominent invader included in: W - 100 of the World's Worst Invasive Alien Species: A Selection from the Global Invasive Species Database ([Lowe, Browne, Boudjelas & De Poorter, 2000](#_ENREF_2)); D - Species Accounts of 100 of the Most Invasive Alien Species in Europe ([DAISIE partners, 2009](#_ENREF_1)) and EU - EU Regulation No. 1143/2014 and Commission Implementing Regulation No. 2016/1141.

1. **All alien animals**

*Acheta domestica*

*Aglossa caprealis*

*Ahasverus advena*

*Alphitophagus bifasciatus*

*Ameiurus nebulosus*

*Ammotragus lervia*

*Amphiareus obscuriceps*

*Anguilla anguilla*

*Anguillicola crassus* D

*Aphis forbesi*

*Aphis oenotherae*

*Apomyelois ceratoniae*

*Appendiseta robiniae*

*Argyresthia thuiella*

*Arion lusitanicus* D

*Aristichthys nobilis*

*Arocatus longiceps*

*Arytaina genistae*

*Ascaridia dissimilis*

*Astacus leptodactylus*

*Attagenus smirnovi*

*Brachyunguis tamaricis*

*Cacopsylla hippophaes*

*Cadra calidella*

*Cameraria ohridella* D

*Capra aegagrus*

*Capreolus pygargus*

*Carassius gibelio*

*Carassius langsdorfii*

*Carpophilus dimidiatus*

*Carpophilus hemipterus*

*Carpophilus ligneus*

*Carpophilus marginellus*

*Carpophilus mutilatus*

*Carpophilus truncatus*

*Ceratitis capitata* D

*Cervus elaphus canadensis*

*Cervus elaphus maral*

*Cervus nippon* D

*Channa argus*

*Chondrostoma nasus*

*Chrysodeixis chalcites*

*Chymomyza amoena*

*Coloradoa abrotani*

*Columba livia f. fera*

*Corbicula fluminea* D

*Corcyra cephalonica*

*Coregonus albula*

*Coregonus autumnalis migratorius*

*Coregonus maraena*

*Coregonus peled*

*Corythucha ciliata*

*Craspedacusta sowerbii*

*Cryptolestes capensis*

*Cryptolestes ferrugineus*

*Cryptolestes pusillus*

*Cryptolestes turcicus*

*Ctenopharyngodon idella*

*Cylindroiulus britannicus*

*Cylindroiulus caeruleocinctus*

*Cylindroiulus latestriatus*

*Cyprinus carpio* W

*Dacne picta*

*Dama dama*

*Daphnia ambigua*

*Dasineura gleditchiae*

*Dermestes ater*

*Desmometopa microps*

*Dictyonota fuliginosa*

*Diestrammena asynamora*

*Dinoderus minutus*

*Dreissena polymorpha* D, W

*Drosophila busckii*

*Drosophila hydei*

*Drosophila immigrans*

*Drosophila repleta*

*Drosophila simulans*

*Dugesia tigrina*

*Duponchelia fovealis*

*Elasmotropis testacea testacea*

*Elophila manilensis*

*Emys orbicularis*

*Endrosis sarcitrella*

*Ephestia elutella*

*Ephestia kuehniella*

*Ericaphis wakibae*

*Eriocheir sinensis* D, W, EU

*Eumerus funeralis*

*Eupteryx melissae*

*Fascioloides magna*

*Ferrissia fragilis*

*Gasterosteus aculeatus*

*Glischrochilus quadrisignatus*

*Gnatocerus cornutus*

*Gyraulus parvus*

*Haplotinea incestella*

*Hemidactylus turcicus*

*Hofmannophila pseudospretella*

*Hucho hucho*

*Hydrotaea aenescens*

*Hyphantria cunea*

*Hypophthalmichthys molitrix*

*Ictalurus punctatus*

*Ictiobus cyprinellus*

*Idiopterus nephrelepidis*

*Illinoia azaleae*

*Illinoia lambersi*

*Impatientinum asiaticum*

*Janetiella siskiyou*

*Japananus hyalinus*

*Lama guanicoe*

*Latheticus oryzae*

*Lepomis gibbosus*

*Leptinotarsa decemlineata* D

*Liriomyza huidobrensis* D

*Litargus balteatus*

*Livilla variegata*

*Locusta migratoria*

*Macrosiphoniella sanborni*

*Macrosiphum euphorbiae*

*Macrosiphum ptericolens*

*Meleagris gallopavo*

*Melogona broelemanni*

*Menetus dilatatus*

*Metcalfa pruinosa*

*Micropterus dolomieu*

*Micropterus salmoides* W

*Moina weismanni*

*Mus domesticus*

*Mus musculus* W

*Muscina angustifrons*

*Mustela vison* D

*Mylopharyngodon piceus*

*Myocastor coypus* D, W, EU

*Myzus ascalonicus*

*Myzus cymbalariae*

*Nitidula flavomaculata*

*Nyctereutes procyonoides* D

*Obolodiplosis robiniae*

*Odocoileus virginianus*

*Oinophila v-flava*

*Ondatra zibenthicus* D, EU

*Opogona sacchari*

*Orconectes limosus* EU

*Orsillus depressus*

*Oryctolagus cuniculus*

*Oryzaephilus mercator*

*Oryzaephilus surinamensis*

*Ovis musimon*

*Oxycarenus lavaterae*

*Oxytelus migrator*

*Pacifastacus leniusculus* EU

*Palorus subdepressus*

*Paralipsa gularis*

*Parectopa robiniella*

*Patamopyrgus antipodarum*

*Pectinatella magnifica*

*Pelomyia occidentalis*

*Philonthus spinipes*

*Phloeotribus caucasicus*

*Phyllonorycter issikii*

*Phyllonorycter leucographella*

*Phyllonorycter robiniella*

*Physella acuta*

*Proasellus coxalis*

*Procyon lotor* D, EU

*Pseudorasbora parva* D, EU

*Psylla buxi*

*Pterostichus caspius*

*Ptinus tectus*

*Rattus norvegicus* D

*Rattus rattus* W

*Reesa vespulae*

*Rhopalosiphoninus latysiphon*

*Rhopalosiphum rufulum*

*Rhyzopertha dominica*

*Romanogobio belingi*

*Rupicapra rupicapra*

*Salvelinus alpinus*

*Salvelinus fontinalis* D

*Salvelinus namaycush*

*Sinanodonta woodiana*

*Sitophilus granarius*

*Sitophilus oryzae*

*Sitotroga cerealella*

*Stephantis mododendri*

*Stictocephala bisonia*

*Testudo graeca*

*Testudo hermanni*

*Testudo horsfieldii*

*Thymallus arcticus baicalensis*

*Tinea pallescentella*

*Tinea translucens*

*Tineola bisselliella*

*Trachemys scripta* D, W, EU

*Trachyopella straminea*

*Tribolium destructor*

*Trichophaga tapetzella*

*Trichosiphonaphisd polygonifoliae*

*Trichosurus vulpecula* W

*Trigonogenius globulus*

*Trogoderma granarium* W

*Tuponia elegans*

*Uloborus plumipes*

*Uroleucon erigeronense*

*Varroa destructor*

*Xyleborinus alni*

1. **Black & grey species**

*Ameiurus nebulosus*

*Anguillicola crassus* D

*Arion lusitanicus* D

*Aristichthys nobilis*

*Astacus leptodactylus*

*Cameraria ohridella* D

*Carassius gibelio*

*Carassius langsdorfii*

*Cervus nippon* D

*Corbicula fluminea* D

*Ctenopharyngodon idella*

*Dreissena polymorpha* D, W

*Eriocheir sinensis* D, W, EU

*Fascioloides magna*

*Hyphantria cunea*

*Hypophthalmichthys molitrix*

*Lepomis gibbosus*

*Micropterus salmoides* W

*Mus musculus* W

*Mustela vison* D

*Myocastor coypus* D, W, EU

*Nyctereutes procyonoides* D

*Ondatra zibenthicus* D, EU

*Orconectes limosus* EU

*Ovis musimon*

*Oxycarenus lavaterae*

*Pacifastacus leniusculus* EU

*Procyon lotor* D, EU

*Pseudorasbora parva* D, EU

*Rattus norvegicus* D

*Rattus rattus* W

*Salvelinus fontinalis* D

*Sinanodonta woodiana*

*Trachemys scripta* D, W, EU

*Varroa destructor*

*Rupicapra rupicapra*

1. **Vertebrates**

*Ameiurus nebulosus*

*Ammotragus lervia*

*Anguilla anguilla*

*Aristichthys nobilis*

*Capra aegagrus*

*Capreolus pygargus*

*Carassius gibelio*

*Carassius langsdorfii*

*Cervus elaphus canadensis*

*Cervus elaphus maral*

*Cervus nippon* D

*Channa argus*

*Chondrostoma nasus*

*Columba livia f. fera*

*Coregonus albula*

*Coregonus autumnalis migratorius*

*Coregonus maraena*

*Coregonus peled*

*Ctenopharyngodon idella*

*Cyprinus carpio* W

*Dama dama*

*Emys orbicularis*

*Gasterosteus aculeatus*

*Hemidactylus turcicus*

*Hucho hucho*

*Hypophthalmichthys molitrix*

*Ictalurus punctatus*

*Ictiobus cyprinellus*

*Lama guanicoe*

*Lepomis gibbosus*

*Meleagris gallopavo*

*Micropterus dolomieu*

*Micropterus salmoides* W

*Mus domesticus*

*Mus musculus* W

*Mustela vison* D

*Mylopharyngodon piceus*

*Myocastor coypus* D, W, EU

*Nyctereutes procyonoides* D

*Odocoileus virginianus*

*Ondatra zibenthicus* D, EU

*Oryctolagus cuniculus*

*Ovis musimon*

*Procyon lotor* D, EU

*Pseudorasbora parva* D, EU

*Rattus norvegicus* D

*Rattus rattus* W

*Romanogobio belingi*

*Rupicapra rupicapra*

*Salvelinus alpinus*

*Salvelinus fontinalis* D

*Salvelinus namaycush*

*Testudo graeca*

*Testudo hermanni*

*Testudo horsfieldii*

*Thymallus arcticus baicalensis*

*Trachemys scripta* D, W, EU

*Trichosurus vulpecula* W

1. **Invertebrates**

*Acheta domestica*

*Aglossa caprealis*

*Ahasverus advena*

*Alphitophagus bifasciatus*

*Amphiareus obscuriceps*

*Anguillicola crassus* D

*Aphis forbesi*

*Aphis oenotherae*

*Apomyelois ceratoniae*

*Appendiseta robiniae*

*Argyresthia thuiella*

*Arion lusitanicus* D

*Arocatus longiceps*

*Arytaina genistae*

*Ascaridia dissimilis*

*Astacus leptodactylus*

*Attagenus smirnovi*

*Brachyunguis tamaricis*

*Cacopsylla hippophaes*

*Cadra calidella*

*Cameraria ohridella* D

*Carpophilus dimidiatus*

*Carpophilus hemipterus*

*Carpophilus ligneus*

*Carpophilus marginellus*

*Carpophilus mutilatus*

*Carpophilus truncatus*

*Ceratitis capitata* D

*Chrysodeixis chalcites*

*Chymomyza amoena*

*Coloradoa abrotani*

*Corbicula fluminea* D

*Corcyra cephalonica*

*Corythucha ciliata*

*Craspedacusta sowerbii*

*Cryptolestes capensis*

*Cryptolestes ferrugineus*

*Cryptolestes pusillus*

*Cryptolestes turcicus*

*Cylindroiulus britannicus*

*Cylindroiulus caeruleocinctus*

*Cylindroiulus latestriatus*

*Dacne picta*

*Daphnia ambigua*

*Dasineura gleditchiae*

*Dermestes ater*

*Desmometopa microps*

*Dictyonota fuliginosa*

*Diestrammena asynamora*

*Dinoderus minutus*

*Dreissena polymorpha* D, W

*Drosophila busckii*

*Drosophila hydei*

*Drosophila immigrans*

*Drosophila repleta*

*Drosophila simulans*

*Dugesia tigrina*

*Duponchelia fovealis*

*Elasmotropis testacea testacea*

*Elophila manilensis*

*Endrosis sarcitrella*

*Ephestia elutella*

*Ephestia kuehniella*

*Ericaphis wakibae*

*Eriocheir sinensis* D, W, EU

*Eumerus funeralis*

*Eupteryx melissae*

*Fascioloides magna*

*Ferrissia fragilis*

*Glischrochilus quadrisignatus*

*Gnatocerus cornutus*

*Gyraulus parvus*

*Haplotinea incestella*

*Hofmannophila pseudospretella*

*Hydrotaea aenescens*

*Hyphantria cunea*

*Idiopterus nephrelepidis*

*Illinoia azaleae*

*Illinoia lambersi*

*Impatientinum asiaticum*

*Janetiella siskiyou*

*Japananus hyalinus*

*Latheticus oryzae*

*Leptinotarsa decemlineata* D

*Liriomyza huidobrensis* D

*Litargus balteatus*

*Livilla variegata*

*Locusta migratoria*

*Macrosiphoniella sanborni*

*Macrosiphum ptericolens*

*Macrosiphum euphorbiae*

*Melogona broelemanni*

*Menetus dilatatus*

*Metcalfa pruinosa*

*Moina weismanni*

*Muscina angustifrons*

*Myzus ascalonicus*

*Myzus cymbalariae*

*Nitidula flavomaculata*

*Obolodiplosis robiniae*

*Oinophila v-flava*

*Opogona sacchari*

*Orconectes limosus* EU

*Orsillus depressus*

*Oryzaephilus mercator*

*Oryzaephilus surinamensis*

*Oxycarenus lavaterae*

*Oxytelus migrator*

*Pacifastacus leniusculus* EU

*Palorus subdepressus*

*Paralipsa gularis*

*Parectopa robiniella*

*Patamopyrgus antipodarum*

*Pectinatella magnifica*

*Pelomyia occidentalis*

*Philonthus spinipes*

*Phloeotribus caucasicus*

*Phyllonorycter issikii*

*Phyllonorycter leucographella*

*Phyllonorycter robiniella*

*Physella acuta*

*Proasellus coxalis*

*Psylla buxi*

*Pterostichus caspius*

*Ptinus tectus*

*Reesa vespulae*

*Rhopalosiphoninus latysiphon*

*Rhopalosiphum rufulum*

*Rhyzopertha dominica*

*Sinanodonta woodiana*

*Sitophilus granarius*

*Sitophilus oryzae*

*Sitotroga cerealella*

*Stephantis mododendri*

*Stictocephala bisonia*

*Tinea pallescentella*

*Tinea translucens*

*Tineola bisselliella*

*Trachyopella straminea*

*Tribolium destructor*

*Trichophaga tapetzella*

*Trichosiphonaphisd polygonifoliae*

*Trigonogenius globulus*

*Trogoderma granarium* W

*Tuponia elegans*

*Uloborus plumipes*

*Uroleucon erigeronense*

*Varroa destructor*

*Xyleborinus alni*

1. **Terrestrial**

*Acheta domestica*

*Aglossa caprealis*

*Ahasverus advena*

*Alphitophagus bifasciatus*

*Ammotragus lervia*

*Amphiareus obscuriceps*

*Aphis forbesi*

*Aphis oenotherae*

*Apomyelois ceratoniae*

*Appendiseta robiniae*

*Argyresthia thuiella*

*Arion lusitanicus* D

*Arocatus longiceps*

*Arytaina genistae*

*Ascaridia dissimilis*

*Attagenus smirnovi*

*Brachyunguis tamaricis*

*Cacopsylla hippophaes*

*Cadra calidella*

*Cameraria ohridella* D

*Capra aegagrus*

*Capreolus pygargus*

*Carpophilus dimidiatus*

*Carpophilus hemipterus*

*Carpophilus ligneus*

*Carpophilus marginellus*

*Carpophilus mutilatus*

*Carpophilus truncatus*

*Ceratitis capitata* D

*Cervus elaphus canadensis*

*Cervus elaphus maral*

*Cervus nippon* D

*Chrysodeixis chalcites*

*Chymomyza amoena*

*Coloradoa abrotani*

*Columba livia f. fera*

*Corcyra cephalonica*

*Corythucha ciliata*

*Cryptolestes capensis*

*Cryptolestes ferrugineus*

*Cryptolestes pusillus*

*Cryptolestes turcicus*

*Cylindroiulus britannicus*

*Cylindroiulus caeruleocinctus*

*Cylindroiulus latestriatus*

*Dacne picta*

*Dama dama*

*Dasineura gleditchiae*

*Dermestes ater*

*Desmometopa microps*

*Dictyonota fuliginosa*

*Diestrammena asynamora*

*Dinoderus minutus*

*Drosophila busckii*

*Drosophila hydei*

*Drosophila immigrans*

*Drosophila repleta*

*Drosophila simulans*

*Duponchelia fovealis*

*Elasmotropis testacea testacea*

*Elophila manilensis*

*Endrosis sarcitrella*

*Ephestia elutella*

*Ephestia kuehniella*

*Ericaphis wakibae*

*Eumerus funeralis*

*Eupteryx melissae*

*Fascioloides magna*

*Glischrochilus quadrisignatus*

*Gnatocerus cornutus*

*Haplotinea incestella*

*Hemidactylus turcicus*

*Hofmannophila pseudospretella*

*Hydrotaea aenescens*

*Hyphantria cunea*

*Idiopterus nephrelepidis*

*Illinoia azaleae*

*Illinoia lambersi*

*Impatientinum asiaticum*

*Janetiella siskiyou*

*Japananus hyalinus*

*Lama guanicoe*

*Latheticus oryzae*

*Leptinotarsa decemlineata* D

*Liriomyza huidobrensis* D

*Litargus balteatus*

*Livilla variegata*

*Locusta migratoria*

*Macrosiphoniella sanborni*

*Macrosiphum euphorbiae*

*Macrosiphum ptericolens*

*Meleagris gallopavo*

*Melogona broelemanni*

*Metcalfa pruinosa*

*Mus domesticus*

*Mus musculus* W

*Muscina angustifrons*

*Mustela vison* D

*Myocastor coypus* D, W, EU

*Myzus ascalonicus*

*Myzus cymbalariae*

*Nitidula flavomaculata*

*Nyctereutes procyonoides* D

*Obolodiplosis robiniae*

*Odocoileus virginianus*

*Oinophila v-flava*

*Ondatra zibenthicus* D, EU

*Opogona sacchari*

*Orsillus depressus*

*Oryctolagus cuniculus*

*Oryzaephilus mercator*

*Oryzaephilus surinamensis*

*Ovis musimon*

*Oxycarenus lavaterae*

*Oxytelus migrator*

*Palorus subdepressus*

*Paralipsa gularis*

*Parectopa robiniella*

*Pelomyia occidentalis*

*Philonthus spinipes*

*Phloeotribus caucasicus*

*Phyllonorycter issikii*

*Phyllonorycter leucographella*

*Phyllonorycter robiniella*

*Procyon lotor* D, EU

*Psylla buxi*

*Pterostichus caspius*

*Ptinus tectus*

*Rattus norvegicus* D

*Rattus rattus* W

*Reesa vespulae*

*Rhopalosiphoninus latysiphon*

*Rhopalosiphum rufulum*

*Rhyzopertha dominica*

*Rupicapra rupicapra*

*Sitophilus granarius*

*Sitophilus oryzae*

*Sitotroga cerealella*

*Stephantis mododendri*

*Stictocephala bisonia*

*Testudo graeca*

*Testudo hermanni*

*Testudo horsfieldii*

*Tinea pallescentella*

*Tinea translucens*

*Tineola bisselliella*

*Trachyopella straminea*

*Tribolium destructor*

*Trichophaga tapetzella*

*Trichosiphonaphisd polygonifoliae*

*Trichosurus vulpecula* W

*Trigonogenius globulus*

*Trogoderma granarium* W

*Tuponia elegans*

*Uloborus plumipes*

*Uroleucon erigeronense*

*Varroa destructor*

*Xyleborinus alni*

1. **Aquatic**

*Ameiurus nebulosus*

*Anguilla anguilla*

*Anguillicola crassus* D

*Aristichthys nobilis*

*Astacus leptodactylus*

*Carassius gibelio*

*Carassius langsdorfii*

*Channa argus*

*Chondrostoma nasus*

*Corbicula fluminea* D

*Coregonus albula*

*Coregonus autumnalis migratorius*

*Coregonus maraena*

*Coregonus peled*

*Craspedacusta sowerbii*

*Ctenopharyngodon idella*

*Cyprinus carpio* W

*Daphnia ambigua*

*Dreissena polymorpha* D, W

*Dugesia tigrina*

*Emys orbicularis*

*Eriocheir sinensis* D, W, EU

*Ferrissia fragilis*

*Gasterosteus aculeatus*

*Gyraulus parvus*

*Hucho hucho*

*Hypophthalmichthys molitrix*

*Ictalurus punctatus*

*Ictiobus cyprinellus*

*Lepomis gibbosus*

*Menetus dilatatus*

*Micropterus dolomieu*

*Micropterus salmoides* W

*Moina weismanni*

*Mylopharyngodon piceus*

*Orconectes limosus* EU

*Pacifastacus leniusculus* EU

*Patamopyrgus antipodarum*

*Pectinatella magnifica*

*Physella acuta*

*Proasellus coxalis*

*Pseudorasbora parva* D, EU

*Romanogobio belingi*

*Salvelinus alpinus*

*Salvelinus fontinalis* D

*Salvelinus namaycush*

*Sinanodonta woodiana*

*Thymallus arcticus baicalensis*

*Trachemys scripta* D, W, EU

**REFERENCES**

DAISIE partners (2009). Species Accounts of 100 of the Most Invasive Alien Species in Europe. In. DAISIE (Eds.), Handbook of Alien Species in Europe (pp. 269-374). Dordrecht, Springer Netherlands.

Lowe, S., Browne, M., Boudjelas, S. & De Poorter, M. (2000). *100 of the World's Worst Invasive Alien Species: A Selection from the Global Invasive Species Database*. Auckland, The Invasive Species Specialist Group.
